# DFT Study of Functionalized Benzoxazole-Based D–*π*–A Architectures: Influence of Ionic Fragments on Optical Properties and Their Potential in OLED and Solar Cell Devices

**DOI:** 10.3390/molecules30183737

**Published:** 2025-09-15

**Authors:** Edwin Rivera, Ronal Ceballo, Oscar Neira, Oriana Avila, Ruben Fonseca

**Affiliations:** Optical Spectroscopy and Laser Research Group (GEL), Department of Physics, Universidad Popular del Cesar, Sabanas Campus, Diagonal 21 No. 29-56, Sabanas del Valle, Valledupar 200005, Cesar, Colombia; rceballo@unicesar.edu.co (R.C.); oscarneira@unicesar.edu.co (O.N.); orianaavila@unicesar.edu.co (O.A.); rubenfonseca@unicesar.edu.co (R.F.)

**Keywords:** D–π–A architectures, hyperpolarizability, two-photon absorption, functionalized benzoxazoles, optoelectronic devices

## Abstract

This theoretical work investigates the linear (absorption and emission) and nonlinear (first hyperpolarizability and TPA) optical properties of donor–π–acceptor (D–π–A) molecular architectures based on functionalized benzoxazoles, with potential applications in optoelectronic technologies such as OLEDs and solar cells. Four π-conjugated compounds were studied in the gas phase and in polar (methanol) and nonpolar (toluene) solvents, employing DFT with the B3LYP and CAM-B3LYP functionals and the 6-311++G(d,p) basis set, as implemented in Gaussian and Dalton. The results reveal that the chemical environment induces spectral shifts and modulates the intensity of electronic transitions. In particular, the compound *2-((4-((5-nitro-2-oxo-1,3-benzoxazol-3(2H)-yl)amino)phenyl)methyl)-1,3-benzoxazole* exhibited outstanding behavior in methanol, with a significant increase in dipole moment, polarizability, and first hyperpolarizability (static and dynamic at 1064 nm), reaching a TPA cross-section close to 150 GM. These findings highlight the key role of ionic substituents in tuning the optical response of π-conjugated systems and underscore their potential as functional materials for high-performance light-emitting and energy-conversion devices.

## 1. Introduction

The investigation of linear optical properties, including absorption and fluorescence, plays a pivotal role in the characterization of functional organic materials due to their direct relevance in the design of optoelectronic and energy conversion devices [1,2,3,4,5,6,7,8,9,10,11,12,13,14,15,16,17,18,19,20,21,22,23,24,25,26,27,28,29,30]. In engineering applications, these properties support the development of advanced technologies such as organic light-emitting diodes (OLEDs) [18,21], lasers, field-effect transistors [1], and organic photovoltaics [4]. Moreover, their implementation in biological and medical contexts has enabled significant advances in biosensing, fluorescent labeling, and molecular diagnostics [3,10,17,22].

Nonetheless, linear optical processes are subject to intrinsic limitations when higher spatial resolution or advanced light-matter interactions are required. In microscopy, the diffraction limit imposed by the wavelength of the excitation source restricts image resolution [6,7], while in emissive and photovoltaic systems, optical losses due to scattering and reabsorption reduce device efficiency [18]. In response to these challenges, nonlinear optics (NLO) introduces mechanisms such as harmonic generation, frequency conversion, and multiphoton absorption, offering new possibilities for controlling light at the molecular and nanoscale levels [14,15,16,17,18,19].

Among the key parameters in NLO, the first-order hyperpolarizability is essential for evaluating the molecular response to intense electric fields and is particularly relevant in applications such as optical switching and signal modulation [30]. Additionally, TPA, a third-order nonlinear phenomenon, has gained considerable attention for its applications in three-dimensional imaging, targeted phototherapy, and the development of materials with multiphoton-responsive properties [13]. Theoretical approaches based on DFT have become indispensable in the prediction and interpretation of such optical responses, offering a favorable compromise between computational efficiency and accuracy [9,24].

Recent studies confirm that benzoxazole derivatives with donor–π–acceptor architectures show outstanding nonlinear optical (NLO) responses [31,32]. Introducing pyridyl or nitro fragments enhances electron density and shifts the absorption into the visible region [26,27]. Other reports demonstrate that lengthening the π-spacer increases β almost linearly, providing a straightforward synthetic strategy to optimize the response [28]. In applications, spin-coated films achieve blue-green photoluminescence quantum yields of 75% in OLED devices [1], while surface-functionalised nanoparticles enable tumour bio-imaging with low cytotoxicity and high physiological stability [9,10].

The compound **B.4** was selected because it forms part of a coherent structural series that allows a controlled assessment of how push–pull strength influences the optical properties of benzoxazole cores. **B.1**, a symmetric terphenyl bearing two benzoxazole units, serves as a reference without strong donor or acceptor groups; **B.2** and **B.3** progressively introduce amino (–NH_2_) and nitro (–NO_2_) motifs linked by π-conjugated bridges, thereby increasing charge separation and, consequently, hyperpolarizability. Finally, **B.4** combines two benzoxazole units with a highly electronegative 5-nitro-2-oxo acceptor, representing the maximum electronic asymmetry in the series. This systematic modification with donor and/or acceptor fragments—while preserving the primary aromatic backbone—enables a direct correlation of changes in geometry, UV-vis spectra, fluorescence, hyperpolarizability and two-photon absorption.

In this context, benzoxazole-based π-conjugated systems have emerged as promising candidates due to their structural tunability, electronic stability, and ability to accommodate electron donor and acceptor groups, thereby facilitating push-pull architectures that enhance their nonlinear optical behavior [2,8,12,19]. The favorable spectroscopic and electronic characteristics of these molecules support their inclusion in studies aimed at the design of advanced photonic and functional materials.

## 2. Results and Discussion

### 2.1. Electrostatic Potential Maps

Electrostatic potential maps (Table 1) reveal negative regions (red) around carbonyl or nitro oxygens and positive zones (blue) near donor nitrogens, outlining the electron flow pathway. In **B.1**, the terphenyl symmetry distributes charge evenly, limiting the dipole moment (μ). In **B.2**, the nitrobenzyl anchor creates a pronounced gradient that promotes D–π–A transfer. **B.3** adds a vinyl bridge and a nitro-aniline moiety that intensify charge separation, elevating β and γ. **B.4**, with dual benzoxazole units and a strong 5-nitro-2-oxo acceptor, exhibits the greatest asymmetry and the highest μ, explaining its strong absorption and hyperpolarizability.

### 2.2. Calculation of Molar Absorptivity

The compounds were designed and modeled in their ground state using the B3LYP functional, and subsequently, Time-Dependent (TD)-DFT with the CAM-B3LYP functional was applied to simulate their electronic absorption properties. The molar absorptivity ε is calculated by Gaussian09 using the following expression:

(1) $$\varepsilon \left(\omega \right)=\frac{N_{A}e^{2}\ln10}{4\pi m_{e}c^{2}\varepsilon_{0}}\sum_{i}f_{i}\frac{\Gamma /2}{{\left(\omega -\omega_{0}\right)}^{2}+{\left(\Gamma /2\right)}^{2}}$$

In Equation (1), the frequency-dependent molar absorptivity ε(ω) is obtained as a superposition of Lorentzian functions [27], each associated with an electronic transition. The contribution of each transition is weighted by its oscillator strength $f_{i}$ and line width Γ, and the entire expression is scaled by fundamental constants: the electron mass $m_{e}$, its charge *e*, the vacuum permittivity $\varepsilon_{0}$, Avogadro’s number $N_{A}$, the speed of light *c*, and the logarithmic factor ln10. This spectral representation accurately describes the intensity and position of each transition, reflecting the probability of absorption and the associated energy.

**Figure 1:**
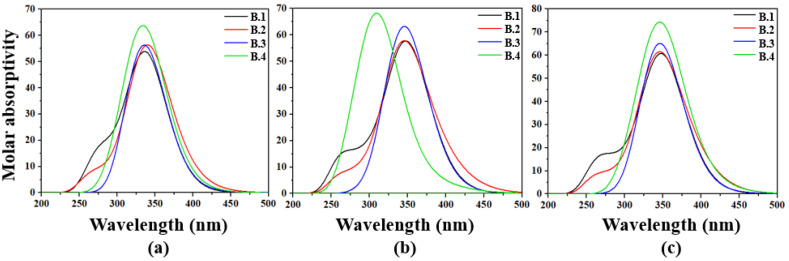
Molar absorptivity spectrum (×1000) calculated for the benzoxazole derivatives. (**a**) Gas phase, (**b**) methanol, and (**c**) toluene.

In the gas phase (Figure 1a), the absorption spectrum of compound **B.1** extends from 240 to 400 nm, covering the ultraviolet and violet regions. It displays two main bands: a more intense one between 276 and 400 nm, dominated by a transition at 338 nm (3.6681 eV, oscillator strength f=1.2726), and a weaker one from 276 to 320 nm, with transitions at 292.95 nm (4.2323 eV, f=0.1953) and 276.64 nm (4.4818 eV, f=0.1798). For derivative **B.2**, the spectrum expands from 240 to 430 nm, with a prominent band from 252.66 to 430 nm and maxima at 377.30 nm (3.2861 eV, f=0.1230), 341.33 nm (3.632 eV, f=1.1596), and 324.58 nm (3.8198 eV, f=0.1963). A weaker band appears between 240 and 310 nm, with peaks at 292.92 nm (4.2328 eV, f=0.1105) and 261.71 nm (4.7375 eV, f=0.1060).

Compound **B.3** exhibits absorption from 290 to 395 nm, with three main transitions at 340.82 nm (3.6378 eV, f=0.7841), 329.86 nm (3.7587 eV, f=0.1269), and 328.89 nm (3.7698 eV, f=0.4972). In contrast, **B.4** spans from 270 to 420 nm with four distinct transitions: 340.82 nm (3.6377 eV, f=1.0079), 332.10 nm (3.7333 eV, f=0.3499), 310.70 nm (3.9905 eV, f=0.2467), and 306.36 nm (4.0470 eV, f=0.1154). These spectral shifts and intensity enhancements demonstrate the impact of electron-donating and electronwithdrawing substituents on the electronic configuration, enabling improved absorption in the near-visible region.

In polar and nonpolar solvents such as methanol and toluene (Figure 1b,c), bathochromic shifts and increased oscillator strengths are observed. In methanol, **B.1** absorbs from 235 to 415 nm with a peak at 349.46 nm (3.5479 eV, f=1.3734); **B.2** shows a maximum at 345.04 nm (3.5934 eV, f=1.1754); **B.3** peaks at 346.91 nm (3.5739 eV, f=1.4754); and **B.4** reaches 351.94 nm (3.5229 eV, f=1.1153), extending up to 600 nm. Similar trends are confirmed in toluene, supporting the compounds’ optical robustness in diverse dielectric environments.

### 2.3. Calculation of Emission

Time-Dependent (TD)-DFT with the CAM-B3LYP functional was applied to simulate their electronic emission properties. To construct the emission spectrum, one begins with the excitations obtained in the absorption study and optimizes the geometry of the first excited state (S_1_). The energy of the emitted photon corresponds to the difference $E_{S_{1}}\left(r_{S_{1}}\right)-E_{S_{0}}\left(r_{S_{1}}\right)$, that is, the electronic energy of the excited state minus that of the ground state, both evaluated at the same relaxed geometry $r_{S_{1}}$. The oscillator strength associated with the downward vertical transition (the radiative probability of returning to the ground state) is then calculated and used in Equation 1 to generate the emission spectrum profile

**Figure 2:**
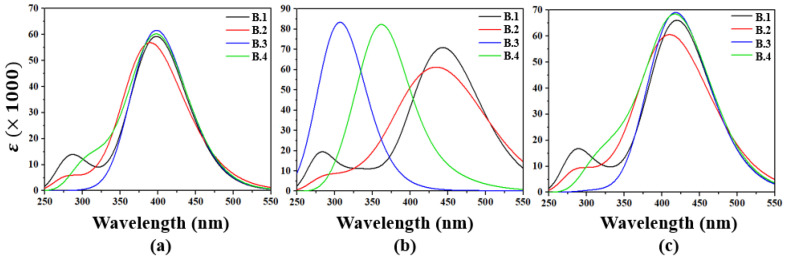
Emission spectrum calculated for the benzoxazole derivatives. (**a**) Gas phase, (**b**) Methanol and (**c**) Toluene.

In the gas phase (Figure 2a), compounds **B.1** to **B.4** exhibit emission profiles with broad bandwidths spanning from the ultraviolet to the edge of the visible region, suggesting their potential as emitters in optoelectronic applications. Compound **B.1** displays a spectrum ranging from 240 to 490 nm, with a dominant band centered at 398 nm (3.1152 eV, *f* = 1.4422), significantly red-shifted relative to its most intense absorption transition at 338 nm (3.6681 eV). This spectral shift indicates a favorable Stokes-type emission. **B.2** extends its emission up to 510 nm, with prominent peaks at 423.38 nm (2.8875 eV) and 385.15 nm (3.2191 eV), both showing notable intensities. Compounds **B.3** and **B.4** also show a redshifted emission trend relative to their absorption bands, with major transitions between 328 and 399 nm and significant oscillator strengths. This separation between absorption and emission may reduce self-absorption processes, enhancing luminous efficiency in devices.

In a polar medium such as methanol (Figure 2b), the emission spectra broaden and exhibit more pronounced bathochromic shifts. **B.1** reaches up to 550 nm, with a maximum at 443.21 nm (2.7974 eV, *f* = 1.7497), higher in both intensity and wavelength compared to the gas phase, reflecting a solvent environment that stabilizes the excited state. For **B.2** and **B.3**, emission maxima appear between 335 and 443 nm, with relatively high intensities (*f* > 0.89). **B.4** shows a spectrum rich in transitions, with at least five significant peaks in the 320–437 nm range. In all cases, the shifts relative to the absorption maxima range from 60 to 100 nm, indicating substantial electronic reorganization between the ground and excited states, promoted by solvent polarity.

In toluene (Figure 2c), a less polar solvent, the general spectral structure observed in methanol is preserved, although slight variations occur in the relative intensities and positions of the maxima. Compounds **B.1** and **B.2** maintain their emission peaks around 443 nm, while **B.3** and **B.4** retain transitions between 320 and 437 nm.

### 2.4. Light-Harvesting Efficiency and High Absorption–Emission Conversion Efficiency

Figure 1 highlights how effectively the four benzoxazole derivatives harvest photons in different media. In the gas phase, **B.4** stands out: its band is the tallest and widest, with a molar absorptivity above 6.0 × 10^4^ L mol^−1^ cm^−1^ from 310 to 360 nm, giving it the highest photon absorption density at the UV-visible edge. **B.3** follows closely, whereas **B.1** and **B.2** show lower peaks that end before 380 nm, capturing less energy in this range.

In methanol, all curves red-shift and intensify. **B.3** now displays the sharpest peak (∼6.0 × 10^4^ L mol^−1^ cm^−1^ at 345 nm), yet B.4 maintains a broad profile extending to 600 nm, capturing part of the solar green region and offsetting its slight loss of maximum height. In toluene, **B.4**’s absolute supremacy re-emerges: its band surpasses 7.0 × 10^4^ L mol^−1^ cm^−1^ while retaining the breadth observed in polar solvents. This spectral stability against dielectric changes confirms that **B.4**’s extended conjugation and push-pull architecture confer the greatest overall light-harvesting efficiency, followed by the intense but narrower absorption of **B.3**. **B.1** and **B.2** act as complementary absorbers, useful for reinforcing specific spectral regions depending on the application.

The four derivatives exhibit greater absorption intensity than the parent molecule and shift their maxima toward the UV–violet region, indicating more extensive electronic conjugation and efficient excitonic coupling. In the gas phase (see Table 2), compounds **B.1**, **B.3**, and **B.4** show band-to-band overlaps below 35%, which minimizes internal reabsorption and allows the captured light energy to be released with minimal losses, making them ideal candidates for emissive devices (OLEDs). Derivative **B.2**, with a 53% overlap, absorbs strongly but reabsorbs part of its own emission, thereby reducing its radiative efficiency.

In methanol (see Table 2), the polar solvent broadens all spectra and increases the overlap; even so, the parent compound remains below 20%, while derivatives **B.1**, **B.3**, and **B.4** reach 50%, 70%, and 95%, respectively. These high percentages favor a reabsorption–reemission cascade and enhance the probability of capturing photons across the entire UV–green range-a desirable feature for active layers in heterojunction solar cells. In toluene (see Table 2), the trend is similar: the parent molecule and derivative B.3 (25% and 34%) maintain high radiative efficiency, whereas B.2 and B.4 (40–50%) sacrifice OLED performance but maximize continuous light harvesting, which is especially useful when the goal is to extend the absorption window into the visible.

### 2.5. Calculation of Dipole Moment, Polarizability, and First Hyperpolarizability

To evaluate the first-order nonlinear optical response under dynamic conditions, time-dependent (TD)-DFT was employed using the CAM-B3LYP functional and the 6-311++G(d,p) [36] basis set, with calculations performed specifically at the wavelength of 1063 nm. The tensor components of β(−2ω;ω,ω) were obtained directly from the “Second Hyperpolarizability” section of the Gaussian09 output file [29], which reports frequency-dependent hyperpolarizability values.

In isotropic media such as solutions or amorphous materials, the measurable nonlinear response through techniques like Hyper–Rayleigh Scattering (HRS) can be approximated using an orientational average of the hyperpolarizability tensor [27], described by Equation (2):

(2) $$\langle \beta_{\mathrm{eff}}^{2}\rangle =\frac{1}{15}\sum_{i,j}{\left(\sum_{k}\beta_{ijk}\right)}^{2}$$

From this averaged quantity, the effective first hyperpolarizability is defined by Equation (3):

(3) $$\beta_{\mathrm{eff}}=\sqrt{\langle \beta_{\mathrm{eff}}^{2}\rangle }$$

The following shows the polarizability (α) and first hyperpolarizabilities static β(0;0,0) and dynamic β(−2ω;ω,ω) at 1064 nm for benzoxazole-derived organic compounds in gas phase, methanol, and toluene.

Table 3 summarizes the computed values of dipole moment, isotropic polarizability (α), and first-order hyperpolarizabilities, both static β(0;0,0) and dynamic β(−2ω;ω,ω), for benzoxazole-based compounds **B.1** to **B.4** in the gas phase, methanol, and toluene, evaluated at the wavelength of 1064 nm. These properties are critical to assessing the suitability of these systems in optoelectronic and nonlinear optical (NLO) applications, including frequency doubling, electro-optic switching, and light harvesting.

The dipole moment values reflect the degree of molecular asymmetry and the influence of solvent interactions. In the gas phase, compound **B.1** shows the lowest polarity (2.29 D), whereas B.4 exhibits the highest (9.09 D), indicating enhanced intramolecular charge separation. In methanol, all compounds display an increase in dipole moment, reaching up to 13.26 D, due to strong solvent–solute interactions that stabilize the polar excited states. Toluene, being less polar, produces intermediate values (2.80 to 10.88 D), consistent with moderate dielectric effects.

A clear trend of increasing polarizability is observed across the series from **B.1** to **B.4**, associated with the increase in molecular size and conjugation. In the gas phase, values range from 348.39 to 384.65 × 10^−24^ esu. Methanol enhances this response significantly (up to 515.74 × 10^−24^ esu), highlighting its capacity to modulate the electronic cloud through solvation effects. Toluene induces intermediate polarizability enhancements, confirming its role as a nonpolar but polarizable medium.

For nonlinear optical properties, both static and dynamic first hyperpolarizabilities show marked enhancements in solution. Static β(0;0,0) values in the gas phase lie between 7.10 and $13.37\times 10^{-30}$ esu but increase considerably in methanol (21.22–$56.90\times 10^{-30}$ esu), particularly for compound **B.4**. Toluene also leads to intermediate enhancement (13.29–$26.21\times 10^{-30}$ esu), suggesting a consistent solvent-induced nonlinear response. Similarly, dynamic hyperpolarizabilities β(−2ω;ω,ω), relevant for frequency-dependent applications like electro-optic modulation and second-harmonic generation, also increase substantially from gas phase values (5.08–$8.19\times 10^{-30}$ esu) to 10.89–$21.84\times 10^{-30}$ esu in methanol and up to $17.95\times 10^{-30}$ esu in toluene.

### 2.6. Calculation of Two-Photon Absorption (TPA)

TPA is described in Dalton using frequency-dependent quadratic response theory [37,38]. This nonlinear optical process is modeled via the second-order transition tensor ($\delta^{\left(2\right)}$), defined as (Equation (4)):

(4) $$\delta^{\left(2\right)}\propto \sum_{i,j}{\left|\sum_{n}\left(\frac{\langle 0|\mu_{i}\left|n\rangle \langle n\right|\mu_{j}|f\rangle }{E_{n}-E_{0}-\omega }+\frac{\langle 0|\mu_{j}\left|n\rangle \langle n\right|\mu_{i}|f\rangle }{E_{n}-E_{0}-\omega }\right)\right|}^{2}$$

Dalton computes [37] this quantity using TDHF or TDDFT methods, and converts it into a two-photon absorption cross-section (in GM units) via the relation $\sigma^{\left(2\right)}\propto \omega^{2}{\left|\delta^{\left(2\right)}\right|}^{2}$, where ω is the photon frequency [28]. The following shows the two-photon absorption (TPA) cross-section of the benzoxazole derivatives.

**Figure 3:**
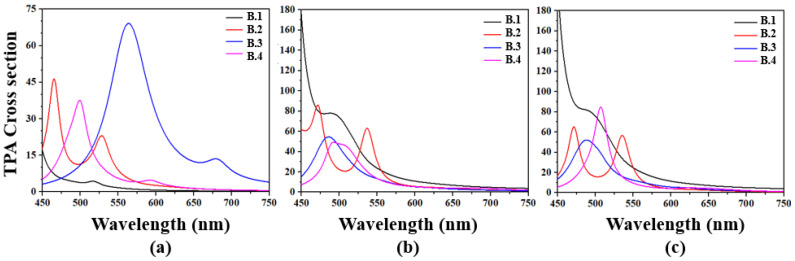
TPA spectrum for the benzoxazole derivatives. (**a**) Gas phase, (**b**) Methanol and (**c**) Toluene.

In the gas phase (Figure 3a), the TPA spectra of compounds **B.1** to **B.4** exhibit well-defined spectral profiles, with maxima located between 475 and 550 nm. Compound **B.1** shows a moderate response, with a peak around 15 GM at 475 nm (2.61 eV), while **B.2** increases its efficiency to approximately 30 GM near 500 nm (2.48 eV). Compound **B.3** stands out for its strong nonlinear response, reaching 70 GM at 550 nm (2.25 eV), accompanied by a secondary signal at 690 nm (1.80 eV), suggesting the presence of additional low-energy transitions. **B.4** exhibits a peak of 35 GM at 520 nm (2.38 eV). The general drop in absorption cross-sections below 20 GM beyond 550 nm indicates an optimal operation window within the visible range, with limited efficiency toward the near-infrared region in this phase.

In a polar medium such as methanol (Figure 3b), a considerable enhancement of the TPA cross-sections is observed, attributed to solvent-induced stabilization of the excited state. **B.1** reaches a maximum value of approximately 160 GM at 470 nm (2.64 eV), while **B.2** and **B.3** display peaks of 85 GM at 500 nm (2.48 eV) and 70 GM at 510 nm (2.43 eV), respectively. B.4 shows a peak of 60 GM at 490 nm (2.53 eV). These enhanced responses in polar medium reinforce the potential of these compounds as active materials in nonlinear optical devices operating under visible excitation, such as multiphoton bioimaging systems or high-sensitivity optical switches.

In toluene (Figure 3c), a less polar solvent, the spectral profiles are largely preserved, though with slightly adjusted intensities. **B.1** retains the highest performance with a cross-section of 170 GM at 480 nm (2.58 eV), followed by **B.2** with 95 GM at 500 nm (2.48 eV), **B.3** with 80 GM at 525 nm (2.36 eV), and **B.4** with 75 GM at 505 nm (2.46 eV). Beyond 550 nm, the absorption cross-sections drop below 15 GM, reaffirming the most efficient spectral window between 470 and 530 nm.

### 2.7. Discussion

The present study quantitatively evaluated the linear and nonlinear optical properties of four donor–π–acceptor (D–π–A) molecular architectures derived from benzoxazole using DFT and TD-DFT. The B3LYP and CAM-B3LYP functionals combined with the 6-311+G(d) basis set were employed to accurately describe both ground and excited electronic states. The results show that the incorporation of electron-donating and electron-withdrawing groups significantly alters the permanent dipole moments (up to 13.26 D) and first hyperpolarizabilities (static β of up to $56.90\times 10^{-30}$ esu), indicating an enhanced nonlinear optical response induced by substitution patterns and solvent polarity.

The computed absorption and emission spectra exhibit bathochromic shifts and variations in transition intensities depending on the medium, with more pronounced effects observed in methanol. The derivatives intensify absorption, shifting maxima to UV–violet. **B.1**, **B.3**, and **B.4** emit efficiently; **B.2** reabsorbs. Methanol broadens spectra and enhances photon capture; toluene preserves trends, overall. Additionally, the two-photon absorption (TPA) cross-sections reached values above 160 GM, particularly for compound **B.1** in polar solvents, positioning these systems as promising candidates for multi-photon bioimaging and active optoelectronic devices. These findings support the rational design of organic materials with dual absorption–emission functionality optimized for photovoltaic technologies and nonlinear optics.

## 3. Computational Methods

Compounds B.1 to B.4 (Table 4) were designed using GaussView [33] and feature benzoxazole cores functionalized with D-π-A (donor-bridge-acceptor) architectures, ideal for organic solar cell applications. B.1 is a symmetric terphenyl bearing two benzoxazole groups at the 4,4’-positions, acting as π-conjugated bridges. B.2 incorporates a nitrobenzylidene acceptor and a benzoxazole-based donor. B.3 includes a vinyl bridge and a N-(4-nitrophenyl)aniline moiety, enhancing electronic asymmetry. B.4 features dual benzoxazole units and a strong 5-nitro-2-oxo acceptor, promoting charge separation. These molecular systems exhibit key photoelectronic properties for solar devices, including efficient light harvesting and intramolecular charge transfer [4,11].

The computational methodology was developed in four stages using DFT at the B3LYP/6-311+G(d) level [36]. Molecular design and optimisation were carried out with Gaussian 09 [34]. Four benzoxazole-based organic systems, labelled **B.1** to **B.4**, were constructed, each displaying donor–acceptor (D–A) architectures. Compound **B.1** corresponds to 4,4’-bis(benzoxazol-2-yl)terphenyl, whereas **B.2** to **B.4** are derivatives that incorporate amino ( –NH_2_ ) groups as electron donors and nitro (–NO_2_) groups as electron acceptors, thereby enhancing push–pull behaviour. All structures were optimised both in the gas phase and in methanol and toluene; moreover, solute–solvent interactions during geometry optimisations and TD-DFT calculations were treated with the **SMD** model (parametrised universal continuum), which includes specific cavity, dispersion and repulsion contributions. Ground states were characterised, UV–Vis absorption and fluorescence spectra were simulated, and static and dynamic first-order hyperpolarisabilities at 1064 nm were calculated. Finally, two-photon absorption (TPA) properties were modelled in Dalton [37] and compared to evaluate their potential for photonic and photovoltaic applications.

## 4. Conclusions

This study demonstrates the potential of benzoxazole-based D–π–A organic architectures as efficient materials for nonlinear optical (NLO) applications. By using DFT and TD-DFT calculations at the B3LYP and CAM-B3LYP levels, it was shown that structural modifications via electron-donating and -withdrawing groups significantly enhance both linear and nonlinear optical responses. Dipole moments increased notably in polar environments, and first-order hyperpolarizabilities—both static and dynamic—were amplified in solution, particularly in methanol, where values reached up to $56.90\times 10^{-30}$ esu for β(0;0,0).

The absorption and emission spectra revealed large Stokes shifts and solvent-sensitive transitions, with red shifts and increased oscillator strengths in more polar solvents. These properties suggest reduced reabsorption and increased quantum yield—key factors in optoelectronic device efficiency. Moreover, the TPA cross-sections exceeded 160 GM under certain conditions, confirming the strong nonlinear optical response and highlighting the feasibility of these systems for multi-photon applications.

Altogether, the results support the rational design of benzoxazole-based compounds as multifunctional chromophores for use in organic photovoltaics, fluorescence imaging, and all-optical switching technologies, where simultaneous absorption and emission control, and strong two-photon responses are essential.

## Figures and Tables

**Table 1:** Electrostatic potential maps of the benzoxazole-based compounds.

Abbr.	Structure	Description
**B.1**	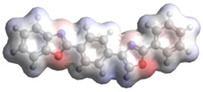	4,4’-bis(benzoxazol-2-yl)terphenyl
**B.2**	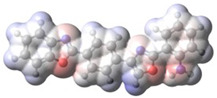	4-((2-(benzoxazol-2-yl)phenyl)amino)-3-nitrobenzylidene)benzoxazole
**B.3**	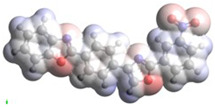	(E)-4-[2-(benzoxazol-2-yl)vinyl]-N-(4-nitrophenyl)aniline
**B.4**	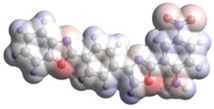	2-((4-((5-nitro-2-oxo-1,3-benzoxazol-3(2H)-yl)amino)phenyl)methyl)-1,3-benzoxazole

The benzoxazole derivatives were designed in GaussView [33], optimized in Gaussian09 [34], and the electrostatic potential map in Avogadro [35].

**Table 2:** Band-to-band overlap of the compounds in different media.

Compound	Medium/Phase	Band-to-Band Overlap (%)
B.1	Gas phase	<35
B.1	Methanol	—
B.1	Toluene	—
B.2	Gas phase	53
B.2	Methanol	50
B.2	Toluene	40–50
B.3	Gas phase	<35
B.3	Methanol	70
B.3	Toluene	34
B.4	Gas phase	<35
B.4	Methanol	95
B.4	Toluene	40–50

**Table 3:** Properties of polarizability (α) and static β(0;0,0) and dynamic β(−2ω;ω,ω) first hyperpolarizabilities at 1064 nm for benzoxazole-derived organic compounds in gas phase, methanol, and toluene.

Organic Compound	Dipole Moment (Debye)	α$\;\times \;10^{-24}$ esu	β(0;0,0)$\;\times \;10^{-30}$ esu	β(−2ω;ω,ω) $\times 10^{-30}$ esu
**Gas Phase**				
**B.1**	2.29	348.39	7.10	5.08
**B.2**	3.82	362.61	13.37	8.19
**B.3**	6.75	367.77	8.34	6.65
**B.4**	9.09	384.65	12.48	8.17
**Methanol**				
**B.1**	3.52	464.39	21.22	10.89
**B.2**	5.73	476.85	31.56	14.74
**B.3**	9.52	497.33	22.33	9.80
**B.4**	13.26	515.74	56.90	21.84
**Toluene**				
**B.1**	2.80	406.47	13.29	9.92
**B.2**	4.59	412.12	20.86	14.93
**B.3**	7.98	429.88	13.56	10.15
**B.4**	10.88	440.36	26.21	17.95

The data were obtained from the .out files by the authors.

**Table 4:** Benzoxazole-based compounds designed in GaussView [30] for photovoltaic applications.

Abbr.	Structure	Description
**B.1**	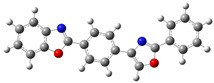	4,4’-bis(benzoxazol-2-yl)terphenyl
**B.2**	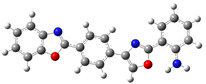	4-((2-(benzoxazol-2-yl)phenyl)amino)-3-nitrobenzylidene)benzoxazole
**B.3**	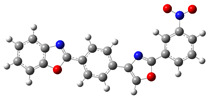	(E)-4-[2-(benzoxazol-2-yl)vinyl]-N-(4-nitrophenyl)aniline
**B.4**	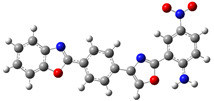	2-((4-((5-nitro-2-oxo-1,3-benzoxazol-3(2H)-yl)amino)phenyl)methyl)-1,3-benzoxazole

Figures in the tables were designed in Avogadro [35] and imported into GaussView [33]. In the ball-and-stick models used, each atom is identified by the standard color scheme: hydrogens appear in white, carbons in dark gray to highlight the aromatic framework, nitrogens in bright blue, and oxygens in red.

## Data Availability

The data supporting the findings of this study, including optimized structures, electronic properties, and nonlinear optical responses, were obtained through quantum chemical calculations using Gaussian software. These data are available from the corresponding author upon reasonable request.

## Abbreviations

The following abbreviations are used in this manuscript:

DFT	Density Functional Theory
TPA	Two-photon absorption
b3lyp	Becke, 3-parameter, Lee–Yang–Parr functional
